# Investigation of a Thermoresponsive In Situ Hydrogel Loaded with Nanotriphala: Implications for Antioxidant, Anti-Inflammatory, and Antimicrobial Therapy in Nasal Disorders

**DOI:** 10.3390/gels11020106

**Published:** 2025-02-02

**Authors:** Rungsinee Phongpradist, Chuda Chittasupho, Sudarshan Singh, Julalak Chorachoo Ontong, Sarin Tadtong, Puriputt Akachaipaibul, Charatda Punvittayagul, Kriangkrai Thongkorn, Pornngarm Dejkriengkraikul, Jutamas Jiaranaikulwanitch, Sunee Chansakaow, Darunee Hongwiset

**Affiliations:** 1 Department of Pharmaceutical Sciences, Faculty of Pharmacy, Chiang Mai University, Muang, Chiang Mai 50200, Thailand; rungsinee.p@cmu.ac.th (R.P.); chuda.c@cmu.ac.th (C.C.); jutamas.jia@cmu.ac.th (J.J.); sunee.c@cmu.ac.th (S.C.); 2 Faculty of Pharmacy, Chiang Mai University, Muang, Chiang Mai 50200, Thailand; sudarshan.s@cmu.ac.th; 3 Office of Research Administration, Chiang Mai University, Muang, Chiang Mai 50200, Thailand; 4 Cosmetic Technology and Dietary Supplement Products Program, Faculty of Agro and Bio Industry, Thaksin University, Ban Pa Phayom, Phatthalung 93210, Thailand; julalak.o@tsu.ac.th; 5 Department of Pharmacognosy, Faculty of Pharmacy, Srinakharinwirot University, Ongkharak, Nakhon Nayok 26120, Thailand; sarin@g.swu.ac.th; 6 Faculty of Pharmacy, Srinakharinwirot University, Ongkharak, Nakhon Nayok 26120, Thailand; puriputt.akachaipaibul@g.swu.ac.th; 7 Center of Veterinary Medical Diagnostic and Animal Health Innovation, Faculty of Veterinary Medicine, Chiang Mai University, Muang, Chiang Mai 50100, Thailand; charatda.pun@cmu.ac.th; 8 Faculty of Veterinary Medicine, Chiang Mai University, Muang, Chiang Mai 50100, Thailand; kriangkrai.th@cmu.ac.th; 9 Department of Biochemistry, Faculty of Medicine, Chiang Mai University, Muang, Chiang Mai 50200, Thailand; pornngarm.d@cmu.ac.th; 10 Center for Research and Development of Natural Products for Health, Chiang Mai University, Muang, Chiang Mai 50200, Thailand; 11 Anticarcinogenesis and Apoptosis Research Cluster, Faculty of Medicine, Chiang Mai University, Muang, Chiang Mai 50200, Thailand

**Keywords:** in situ hydrogel, triphala, nasal delivery pathway, antioxidant, antiinflammation, antimicrobial, nanoparticles, nasal disorder

## Abstract

Oxidative stress plays a crucial role in chronic nasal disorders, contributing to inflammation, tissue damage, and impaired mucosal function, highlighting the need for targeted therapies. Recent advancements in nasal drug delivery systems have expanded their applications for treating respiratory and inflammatory conditions. Among these, hydrogel-based systems offer prolonged release of active pharmaceutical ingredients (APIs), enhancing therapeutic efficacy and reducing dosing frequency. This study initially evaluates the antioxidant, anti-inflammatory, antimicrobial, and cytotoxic properties of Nanotriphala, followed by its incorporation into a thermoresponsive in situ hydrogel system, which was subsequently developed and characterized as a novel formulation. Nanotriphala exhibited >90% cell viability and significantly reduced nitric oxide (NO) levels by 40.55 µg/mL at 250 µg/mL. The hydrogel was characterized by key parameters, including viscosity, gelling time, pH, gelling temperature, texture analysis, and ex vivo spreadability. Stability was assessed under various conditions, and mutagenicity and antimutagenicity were evaluated using the Ames test. Results showed that the hydrogel gelled at 34 °C, exhibited good spreadability (10.25 ± 0.28 cm), a viscosity of 227 ± 22 cP, and maintained a pH of 5.75 ± 0.01, with optimal hardness and adhesiveness suitable for nasal application. It demonstrated antimicrobial activity against *E. coli*, *P. aeruginosa*, *S. aureus*, and *S. epidermidis* at minimal bactericidal concentrations (MBCs) of 32, 2, 4, and 8 µg/mL, respectively, with low mutagenicity (mutagenic index < 2) and strong antimutagenic activity (>60%). The gallic acid content was 0.5796 ± 0.0218 µg/100 mL. Stability studies confirmed optimal storage at 4 °C. These findings suggest that in situ hydrogel loaded with Nanotriphala is a promising nasal drug delivery system for managing oxidative stress and related inflammatory conditions.

## 1. Introduction

Nasal disorders, such as allergic rhinitis, chronic sinusitis, and rhinosinusitis, have a significant burden on individual health and healthcare systems. These conditions are characterized by chronic inflammation, tissue damage, and impaired mucociliary clearance, which are often exacerbated by oxidative stress [1,2]. Oxidative stress occurs due to an imbalance between the production of reactive oxygen species (ROS) and the antioxidant defenses, playing a pivotal role in the pathogenesis and progression of these disorders [3]. Chronic inflammation and oxidative damage contribute to the ongoing nature of nasal disorders, underscoring the need for innovative treatments that address these core issues. Current nasal therapies, including corticosteroids and antihistamines, effectively alleviate symptoms and reduce oxidative stress. When used together, these treatments amplify their effects, leading to a notable reduction in total oxidative stress and offering a more targeted approach to managing these conditions [4,5]. However, systemic treatments may cause undesired side effects, emphasizing the importance of localized therapies that deliver relief directly to the nasal cavity. In this light, recent advancements in drug delivery systems designed for sustained and localized release of medications, have drawn considerable attention [6,7].

Triphala is a polyherbal formulation composed of three plants: *Emblica officinalis*, *Terminalia chebula*, and *Terminalia bellerica.* It is traditionally prepared with equal parts of *E. officinalis*, *T. chebula*, and *T. bellerica.* However, a modified formulation with varying ratios of *T. chebula*, *T. bellerica*, and *E. officinalis* has been reported to optimize the therapeutic effects of Triphala, enabling targeted benefits tailored to specific health concerns [8,9]. This herbal combination is rich in bioactive compounds, including polyphenols (gallic acid, ellagic acid, chebulinic acid, chebulagic acid, and emblicanin A and B), flavonoids (quercetin and kaempferol), and tannins, which provide strong antioxidant, anti-inflammatory, and antimicrobial effects [10,11,12,13]. Chemical analysis of Triphala reveals numerous biologically active constituents, including chebulinic acid, gallic acid, rutin, quercetin, tannins, flavonoids, pectin, and vitamin C, which collectively contribute to its potent antioxidant and health-promoting effects [10]. The amount of each phytochemical is influenced by the extraction techniques utilized, the type of solvents applied, and the specific geographic location where the parent plant is grown [13]. Pharmacological studies have demonstrated that Triphala extract possesses potent antioxidant activity, helping to reduce damage caused by oxidative stress [9]. This has led to its growing recognition as a potential remedy for oxidative stress-related disorders. Despite its therapeutic potential, the clinical use of Triphala is often hindered by challenges related to the poor solubility, stability, and bioavailability of its active components [14]. To overcome these limitations, advancements in nanotechnology and modern drug delivery systems offer a promising approach to enhance its efficacy and application.

Nanocarrier systems have been shown to enhance the solubility, stability, and bioavailability of herbal extracts, leading to improved therapeutic outcomes [15]. Among these, thermoresponsive in situ hydrogels have emerged as innovative solutions for nasal drug delivery [16]. These hydrogels exhibit a sol-to-gel transition when exposed to physiological temperatures, enabling convenient administration in liquid form followed by gelation within the nasal cavity [17]. This transition not only ensures prolonged retention and controlled release of encapsulated therapeutic agents but also enhances local efficacy while reducing systemic exposure [18]. Advancements in nanotechnology-based delivery systems have shown notable success in improving the bioavailability and therapeutic effectiveness of herbal extracts [19]. Similarly, nanoformulations of other herbal products have demonstrated enhanced penetration through biological barriers, leading to better therapeutic results in preclinical studies. These developments highlight the potential of integrating nanotechnology with traditional herbal therapies to overcome limitations and optimize clinical outcomes [20,21].

The development of a thermoresponsive in situ hydrogel loaded with Nanotriphala (In situ Tri) represents a novel approach to leverage the therapeutic potential of this traditional formulation for treating nasal disorders. By merging the benefits of nanotechnology with the unique properties of in situ gelling systems, this approach aims to overcome the limitations associated with conventional formulations of herbal extracts. A thorough evaluation of the hydrogel’s physicochemical properties, antioxidant capacity, anti-inflammatory effects, cytotoxicity profile, and antimicrobial effectiveness against clinically relevant pathogens will be critical to validate its potential. Furthermore, safety assessment remains a cornerstone of pharmaceutical development. The Salmonella/human S9 mutagenicity test, a well-established method for assessing the genotoxicity of compounds, provides vital information about the safety profile of the developed formulation [22]. This comprehensive framework for characterization and safety evaluation not only ensures alignment with regulatory standards but also underscores adherence to best practices in modern pharmaceutical development.

Merging traditional herbal medicine with advanced drug delivery technologies offers valuable insights into enhancing the efficacy and safety of herbal formulations, bridging traditional knowledge with modern pharmaceutical advancements. Thus, incorporating Triphala extract into a nanoformulation and combining it with an in situ hydrogel could be a promising strategy to overcome the limitations of traditional Triphala formulations for nasal application. The nanoformulation will enhance the solubility, stability, and bioavailability of Triphala’s active components [23], addressing challenges such as poor absorption and rapid degradation, as mentioned earlier. Integrating this nanoformulation into a thermoresponsive in situ hydrogel could amplify its benefits by enabling sustained and localized drug release within the nasal cavity [24]. This dual approach not only improves the therapeutic efficacy of Triphala but also reduces systemic exposure, thereby minimizing potential side effects. Consequently, the combined system may promote the potential to maximize the antioxidant, anti-inflammatory, and antimicrobial activities of Triphala, making it a highly effective treatment option for oxidative stress-related nasal disorders.

This study explores the development of a thermoresponsive in situ hydrogel incorporating Nanotriphala, with the goal of evaluating its biological and pharmacological properties. The research focuses on the antioxidant activity, anti-inflammatory effects, cytotoxicity, and antimicrobial efficacy of Nanotriphala. The antioxidant activity was assessed using established assays, such as DPPH, ABTS, and FRAP. The anti-inflammatory effect was quantified through nitric oxide assays, while cytotoxicity was evaluated using MTT assays to analyze the compound’s impact on cell viability. The antimicrobial potential was tested against key pathogens, including *Escherichia coli*, *Pseudomonas aeruginosa*, *Staphylococcus aureus*, and *Staphylococcus epidermidis*. Subsequently, Nanotriphala was integrated into a thermoresponsive in situ hydrogel, which undergoes detailed characterization of its physicochemical properties, including gelation behavior, viscosity, and thermal stability. The stability of the hydrogel formulation was also evaluated under various storage conditions. To ensure safety, the genotoxic potential of in situ hydrogel loaded with Nanotriphala was analyzed using the Salmonella/human S9 mutagenicity test.

This research aims to uncover the therapeutic potential and safety of this innovative hydrogel system for managing oxidative stress-related nasal disorders. The outcomes may contribute to the development of novel treatment options that bridge traditional knowledge and modern pharmaceutical science.

## 2. Results and Discussion

### 2.1. Antioxidant Activities of Triphala Extract and Nanotirphala

**Figure 1:**
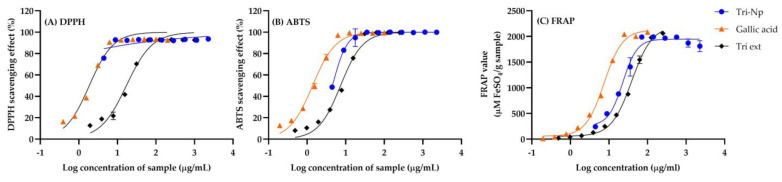
The antioxidant activity of gallic acid, Triphala extract, and Nanotriphala as determined by (**A**) DPPH free radical-scavenging assay, (**B**) ABTS free radical-scavenging assay, and (**C**) ferric reducing antioxidant power (FRAP) assay. Data are presented as mean ± SD (n = 3).

The assessment of antioxidant activity for gallic acid, Triphala extract (Tri ext), and Nanotriphala (Tri Np) using DPPH, ABTS, and FRAP assays provides crucial insights into the potential therapeutic benefits of these compounds in combating oxidative stress. For the DPPH assay (Figure 1A), gallic acid, a standard antioxidant compound, exhibited an IC_50_ value of 1.863 ± 0.191 µg/mL. In contrast, Triphala extract demonstrated higher IC_50_ values of 17.07 ± 2.02 µg/mL, indicating relatively lower antioxidant efficiency compared to gallic acid. However, Nanotriphala displayed lower IC_50_ values of 0.004 ± 0.111 µg/mL, demonstrating a significant increase in free radical-scavenging capacity for the nanoformulation. This significant improvement in antioxidant activity can be attributed to nanoformulation, which enhances the solubility of the active compounds in Triphala. This observation aligns with findings from our previous study, which demonstrated that nanoformulation improves the solubility of Triphala extract [23]. The reduced particle size in Nanotriphala likely facilitates better interaction with DPPH radicals, resulting in improved radical-scavenging efficiency. This observation aligns with a previous report, which highlighted that the nanoscale dimensions of nanoparticles play a critical role in amplifying their surface area [25]. The increased surface area facilitates enhanced interactions with free radicals, thereby significantly bolstering their scavenging capabilities. These amplified antioxidant properties are crucial for combating oxidative stress, which plays a significant role in the development of various conditions, including neurodegenerative diseases, cardiovascular disorders, and cancer [26,27].

Furthermore, the ABTS assay (Figure 1B) showed IC_50_ values of 1.383 ± 0.117 µg/mL for gallic acid, 7.343 ± 0.707 µg/mL for Triphala extract, and 4.533 ± 0.211 µg/mL for Nanotriphala. The remarkably low IC_50_ for Nanotriphala confirms its superior ability to scavenge ABTS radicals, which could be attributed to the synergistic action of the diverse phytochemicals in Triphala. This synergy aligns with evidence suggesting that combinations of phytochemicals often outperform individual compounds in antioxidant efficacy, as they target a broader spectrum of oxidative pathways [28,29].

The ferric-reducing antioxidant power (FRAP) of gallic acid, Triphala extract, and Nanotriphala is summarized in Figure 1C. A calibration curve for ferrous sulfate (Fe^2+^) was constructed, demonstrating excellent linearity within the concentration range of 12.5 to 200 μM, with a correlation coefficient (R^2^) of 0.9957. The FRAP values were expressed in terms of Fe^2+^ equivalents. The data indicated a concentration-dependent increase in the FRAP values for gallic acid, Triphala extract, and Nanotriphala. Specifically, the FRAP values for gallic acid ranged from 11.58 ± 6.42 to 2084.22 ± 24.09 µM, from 19.38 ± 2.44 to 2062.00 ± 48.00 µM for Triphala extract, and from 241.75 ± 3.94 to 1812.37 ± 108.15 µM for Nanotriphala. The ferric-reducing antioxidant power (FRAP) results for Triphala extract and Nanotriphala demonstrate a concentration-dependent increase in antioxidant activity. The concentration range for Triphala extract is 0.49–250 μg/mL, while for Nanotriphala, it is 4.43–2272 μg/mL, which is equivalent to the weight of Triphala extract per milliliter. In the case of Triphala extract, the FRAP values ranged from 19.38 ± 2.44 µM to 2062.00 ± 48.00 µM, showing an increase in antioxidant activity as the concentration increased. For Nanotriphala, with concentrations between 4.43 and 2272 μg/mL, the FRAP values ranged from 241.75 ± 3.94 µM to 1812.37 ± 108.15 µM. Despite the wider concentration range of Nanotriphala, its FRAP values were generally higher at lower concentrations than those of Triphala extract, indicating that Nanotriphala provides more efficient or potent antioxidant activity per unit concentration. This enhanced reducing capability emphasizes the formulation’s role in maintaining the stability of antioxidant compounds, critical for their efficacy in biological systems where environmental factors often lead to the breakdown of these compounds, worsening the effects of oxidative stress [30,31].

The notable increase in antioxidant activity observed with the Nanotriphala formulation underscores the potential of nanoparticulate systems to enhance the therapeutic effectiveness of herbal extracts. When compared to Triphala extract, Nanotriphala stands out as a promising innovation for improving the antioxidant benefits of natural compounds. Future research should focus on elucidating the specific mechanisms driving these improvements and exploring the clinical applications of Nanotriphala in treating oxidative stress-related disorders.

### 2.2. Antimicrobial Activities of Triphala Extract and Nanotriphala

The antimicrobial activity results (see Table 1) for Triphala extract (Tri ext), Nanotriphala (Tri Np), and standard antibiotic treatments demonstrate significant differences in efficacy against various bacterial strains associated with oxidative stress-related nasal diseases. These results indicate that while Triphala extract demonstrates moderate antibacterial properties, particularly against *E. coli* and *S. aureus*, its efficacy is limited, as evidenced by high MBC values. The high MIC and MBC for *P. aeruginosa*, a pathogen known for its resistance, suggest that Triphala extract may require multiple doses of treatment for infections caused by this bacterium. In comparison, Nanotriphala exhibited slightly reduced antimicrobial activity compared to the Triphala extract at equivalent concentrations, likely due to the slower release of the active ingredients from the nanoparticle matrix, which may enable prolonged interaction with bacterial cells [11]. However, Nanotriphala demonstrated activity against *P. aeruginosa*, achieving MIC and MBC values of 12,500 µg/mL and 25,000 µg/mL, respectively; this suggests potential utility for Nanotriphala in treating resistant bacterial infections. While the MIC and MBC values for both Tri ext and Tri Np were higher than those of standard antibiotics, this indicates limited efficacy compared to established treatments. Standard antibiotics (Gentamicin and Vancomycin) served as controls, displaying low MIC and MBC values (0.5 µg/mL for Gentamicin against *S. aureus* and varying for Vancomycin). The results confirm the effectiveness of these antibiotics. However, the enhanced activity of Nanotriphala suggests that it may serve as a complementary or alternative approach, especially in cases of antibiotic resistance [32]. The findings indicate that Nanotriphala not only enhances the antimicrobial properties of Triphala extract but also provides a promising strategy for addressing oxidative stress-related nasal diseases. This enhanced efficacy could be attributed to the encapsulation of bioactive compounds within nanoparticles or a gel matrix, which may protect them from degradation and facilitate sustained release, thus improving their interaction with bacterial cells [33]. However, further optimization of the formulation, including release kinetics and concentration adjustments, is necessary to enhance its antimicrobial efficacy to levels comparable with standard antibiotics. Previous studies have demonstrated the potential of Triphala as an antimicrobial agent due to its rich polyphenolic content [34,35]. Additionally, the therapeutic potential of Triphala’s as a delivery system for improving the therapeutic index of herbal extracts has been highlighted by Peterson et al., in their review, suggesting that nanoencapsulation may prolong the efficacy, improve bioavailability, and protect bioactive compounds from premature degradation [36]. Thus, iterations of this study could compare activity against multidrug-resistant strains, as seen in other nanoparticle-based antimicrobial formulations. In conclusion, Nanotriphala presents significant potential as an innovative therapeutic strategy for bacterial infections related to oxidative stress in nasal conditions.

### 2.3. Cytotoxicity of Triphala Extract and Nanotriphala

**Figure 2:**
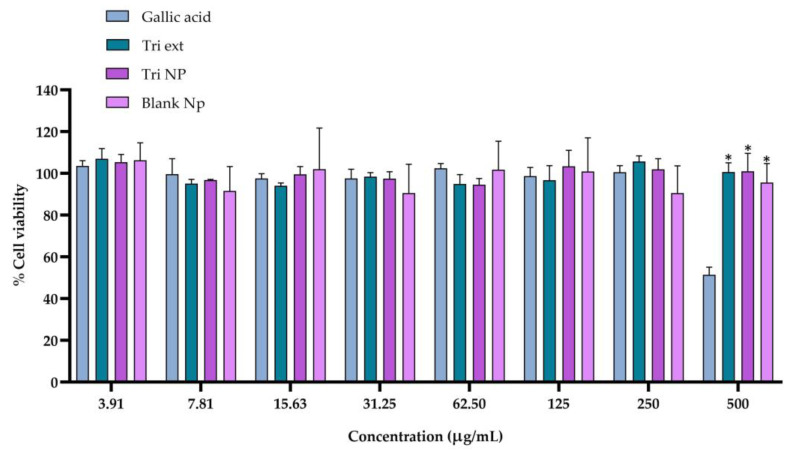
Cell viability (MTT assay) in RAW 267.4 cells cultured on callic acid, Triphala extract (Tri ext), Nanotriphala (Tri Np), and blank nanoparticles (Blank Np). The data represent the mean ± standard deviation (SD) of three independent experiments (n = 3). * indicates a significant difference (*p* < 0.05) when compared to gallic acid at the same concentration.

Due to the importance of ensuring cell viability, the primary purpose of assessing the cytotoxicity of gallic acid, Triphala extract, and Nanotriphala against RAW264.7 cells (used as an anti-inflammatory model) was to establish the maximum non-toxic concentration suitable for the nitric oxide (NO) assay, which serves as a critical evaluation of their anti-inflammatory potential. The results, depicted in Figure 2, show the cell viability percentage, with the control group set at 100%. At 500 μg/mL, gallic acid significantly reduced cell viability, while Triphala extract and Nanotriphala, at concentrations ranging from 3.91 to 500 μg/mL, maintained cell viability above 80%, indicating minimal toxicity [37]. In general, all formulations exhibited favorable cytotoxicity profiles at lower concentrations, with Nanotriphala and Triphala extract maintaining viability above 90% at most doses. Notably, Nanotriphala consistently preserved cell viability at above 94%, even at higher doses, demonstrating excellent biocompatibility. These findings align with previous studies assessing the cytotoxicity of nanoparticles, further supporting the safety and potential of Nanotriphala for biomedical applications [38]. A slight reduction in viability was noted at 500 μg/mL, indicating a threshold beyond which toxicity might occur. While gallic acid showed high cell viability at lower doses, it caused a significant decrease (51.43%) at 500 μg/mL, reflecting dose-dependent cytotoxicity, as noted in previous studies [39]. Blank nanoparticles maintained stable viability at all concentrations, confirming the non-toxic nature of the nanoparticle excipient and its ability to support cell health effectively. Based on these findings, concentrations of 250 μg/mL or lower for gallic acid, Triphala extract, and Nanotriphala were selected for the nitric oxide (NO) assay to ensure cell viability and reliable assessment of their anti-inflammatory potential.

### 2.4. Anti-Inflammatory Activity of Nanotriphala

**Figure 3:**
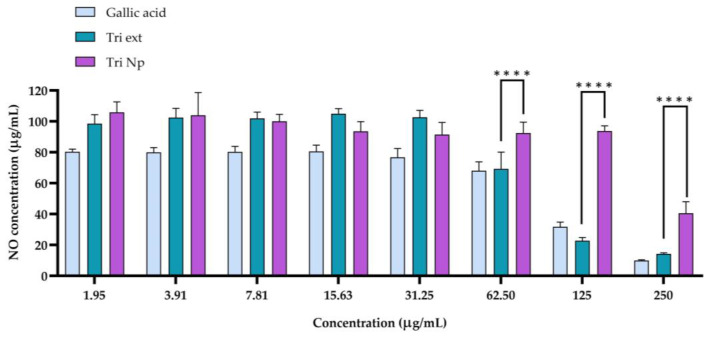
Inhibition of nitric oxide production by gallic acid, Triphala extract, and Nano-Triphala in lipopolysaccharide (LPS)-induced RAW 264.7 macrophages. The data represent the mean ± standard deviation (SD) of three independent experiments (n = 3). **** indicates *p* < 0.0001 when compared with Triphala extract at the same concentration.

The results from the nitric oxide (NO) assay (Figure 3) demonstrate the anti-inflammatory potential of gallic acid, Triphala extract (Tri ext), and Nanotriphala (Tri Np) at various concentrations. Nitric oxide, produced by nitric oxide synthase (NOS), plays a crucial role in inflammation, and its inhibition can indicate anti-inflammatory effects [40]. At lower concentrations (1.953 µg/mL to 31.25 µg/mL), gallic acid caused only a slight reduction in NO levels, which remained close to 80 µg/mL. However, as the concentration increased beyond 62.5 µg/mL, there was a notable decline in NO production. At 250 µg/mL, the NO level was reduced significantly to 9.99 µg/mL. Gallic acid demonstrated the strongest anti-inflammatory effects at higher concentrations, in agreement with its known ability to inhibit pro-inflammatory mediators [41]. This steep decline suggests that gallic acid possesses potent anti-inflammatory activity at higher concentrations, possibly through the inhibition of NOS activity and the reduction in NO production, as shown in previous studies [42,43]. Triphala extract (Tri ext) showed a more gradual decrease in NO production compared to gallic acid. At lower concentrations of Triphala extract (1.953 µg/mL to 31.25 µg/mL), the NO level was still high and ranged from 98 to 104 µg/mL. Contrastingly, a drastic decline in NO levels was seen as the doses reached 125 µg/mL and 250 µg/mL, with NO levels of 22.73 µg/mL and 14.29 µg/mL, respectively. This implies that higher doses of Triphala extract may, therefore, be required to achieve a similar level of NO inhibition as with gallic acid. However, Triphala extract can also have an anti-inflammatory effect. These findings also confirm an earlier study that identified Triphala’s bioactive compounds (such as tannins, flavonoids, and polyphenols) with anti-inflammatory and antioxidant activity [44,45]. The Nanotriphala (Tri Np) formulation demonstrated a unique profile of NO inhibition. At lower concentrations (1.953 µg/mL to 31.25 µg/mL), the NO levels produced by Tri Np were comparable to the control group, demonstrating limited anti-inflammatory activity at these doses. However, NO production began to decrease at concentrations of 62.5 µg/mL and above, with NO levels at 250 µg/mL measuring 40.55 µg/mL. This suggests that Tri Np still exhibited anti-inflammatory properties at higher doses, but the reduction in NO levels was not as significant as that of gallic acid. When comparing the concentration range of 62.5–250 µg/mL between Nanotriphala and Triphala extract, the initial results indicated that Triphala extract significantly reduced NO levels compared to Nanotriphala at the same concentrations. However, when calculating the equivalent concentration of Triphala extract in Nanotriphala, it was found that 62.5, 125, and 250 µg/mL of Nanotriphala correspond to approximately 2.84, 5.68, and 11.36 µg/mL of Triphala extract, respectively. At 2.84 and 5.68 µg/mL, the reduction in NO levels by Nanotriphala was not significantly different from the reductions observed at the nearest higher concentrations of Triphala extract (3.91 and 7.81 µg/mL, respectively). Interestingly, at 250 µg/mL of Nanotriphala, which is equivalent to 11.36 µg/mL of Triphala extract, the NO level reduction was significantly greater when compared to 15.63 µg/mL of Triphala extract. Hence, we demonstrate the anti-inflammatory properties of Nanotriphala by considering the significant reduction in NO generation in inflammatory RAW267.4 macrophage cells. These delivery systems are known to improve the solubility, stability, and therapeutic effectiveness of bioactive components of food, thus increasing their ability to modulate negative inflammatory responses [46].

The results from the nitric oxide assay demonstrate that the anti-inflammatory effect of Tri ext and Tri Np were concentration-dependent. Hence, according to this study, gallic acid and Triphala extract, as well as Nanotriphala, exhibited nitric oxide synthesis inhibition in a concentration-dependent manner, thereby indicating their anti-inflammatory potential. These results highlight the potential application of Nanotriphala as a biocompatible and effective delivery system for anti-inflammatory therapy, especially for diseases associated with oxidative stress.

### 2.5. Physicochemical Properties of In Situ Gel Loaded with Nanotriphala

**Figure 4:**
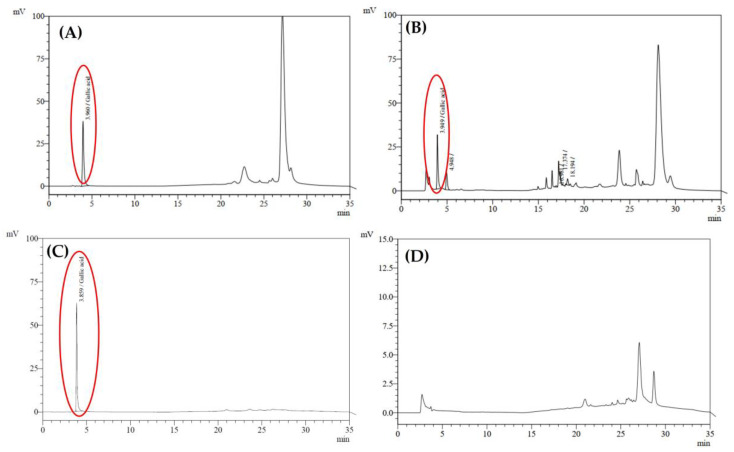
HPLC chromatogram of (**A**) standard gallic acid in blank hydrogel sample, (**B**) gallic acid in in situ hydrogel Nanotriphala, (**C**) gallic acid standard solution, and (**D**) blank in situ hydrogel.

After incorporating Nanotriphala, with a size of 137.6 ± 1.9 nm, a PDI of 0.13 ± 0.01, and a zeta potential of −22.73 ± 0.23 mV, into an in situ hydrogel system, the resulting formulation was characterized for its physicochemical properties. Numerous important inferences related to the properties of the in situ gel comprising nanoparticles with Triphala extract that are useful in treating oxidative stress-mediated nasal disorders are drawn from the characterization results (Table 2). The obtained pH value of 5.75 falls within the physiological values of nasal secretions, which are generally between 5.5 and 6.5 [47,48]. This slightly acidic pH is essential for several reasons. First, it reduces the potential for nasal irritation, a concern specific to topical preparations. Research shows that products with pH above or below the physiological range can cause irritation and even damage to the nasal mucosa [49]. The gelling time of 26.27 ± 1.16 s falls within the suitable range reported in previous studies, indicating that the formulation is well suited for nasal application [50,51,52]. The short gelation time will ensure a prolonged residence time in the nasal cavity, effectively counteracting mucociliary inflammation [53]. A rapid-to-gel phase transition upon administration can effectively prevent draining from the nasal cavity and allow sustained contact with the mucosal surface. This property is consistent with previous results from Ivano et al. [54], who reported that the bioavailability of thermosensitive gels was significantly greater than that of conventional delivery in the case of gels (32–34 °C) with the same gelling temperatures. Moreover, the gelation temperature (34.0 °C) is also well in the range of the human body temperature, which enables the fluid to be smoothly administered and gel quickly upon contact with nasal mucosa [55]. In our previous investigation, gallic acid, chebulagic acid, and chebulinic acid were found to be the major components of the Nanotriphala preparation. Nevertheless, to reflect the actual studies, for this study, gallic acid was chosen as the predominant active compound for its well-known therapeutic effects. The retention time of gallic acid was about 3.960 min (Figure 4A), and the quantification analysis presented a linear response from 3.001 to 9.004 µg/mL with an R^2^ value over 0.99, supporting the precision and reliability of the analytical method. This was coupled with a tailing factor of 1.755 ± 0.040, theoretical plate count of 18,018 ± 377, resolution of 4.092 ± 0.084, and a signal-to-noise ratio of 50.12 ± 38.00, collectively demonstrating excellent system suitability for chromatographic analysis. Gallic acid in In situ Tri (Figure 4B) recorded a retention time of 3.961 ± 0.002 min. The chromatogram of the blank in situ hydrogel (Figure 4D) reveals a major peak around 27 min, likely representing components or impurities inherent to the hydrogel formulation that elute at this retention time. Importantly, this peak does not overlap with the retention time of gallic acid (3.960 min), confirming the method’s selectivity and suitability for the accurate quantification of the gallic acid without interference from the hydrogel matrix. For In situ Tri, temperature control was crucial throughout the sample preparation process. The sample temperature was kept below 30 °C to avoid premature gelation and the column temperature was set at 17 °C to provide optimal separation and accurate determination of the chromatographic analysis. The final concentration of gallic acid in the In situ Tri formulation was determined as 0.5796 ± 0.0218 µg/100 mL, indicating that while the amount of gallic acid achieved in the preparation was not very high, it is nevertheless therapeutically important for the action of intra-nasal therapy. Gallic acid is well known for being a potent antioxidant, anti-inflammatory, and antimicrobial agent, which makes it a promising agent for the treatment of nasal disorders like allergic rhinitis, sinusitis, and other diseases related to oxidative stress, inflammation, and microbial infection [56]. The viscosity of the formulation at 227 cP is likely to lie within the range of optimal nasal gels (200–1000 cP). Such intermediate viscosity is suitable for easy administration but offers sufficient resistance to the mucociliary clearance processes to prolong the contact between the drug and the nasal epithelium [57]. Viscosity and mucoadhesion must be well balanced to guarantee drug absorption and therapeutic effectiveness. A relatively high spreadability value of 10.25 cm was reported as one of the delivery properties of the developed formulations, which guarantees good and even nasal mucus coverage [58]. This value suggests that all the tested formulations have good spreadability and are therefore appropriate to be used nasally, which is in line with the results obtained from previous studies [58]. Adequate viscosity is essential to prolong nasal residence time, ensuring sustained contact with the mucosa for effective drug delivery [58]. Low viscosity allows easy packing, handling, and hassle-free administration of the formulation, while a higher viscosity may accompany a prolonged retention time in the nasal cavity, which also limits the permeation rate. Thus, it is important to achieve optimal viscosity. The viscosity of Nanotriphala in this study was observed to be between 100 and 200 cP, which falls in the range of viscosity that is reported to be optimal for nasal administration [59,60].

Overall, the In situ Tri formulation demonstrates favorable physicochemical properties, such as an appropriate pH, rapid gelling, optimal viscosity, and good spreadability, all of which are crucial for effective nasal drug delivery. These characteristics suggest potential for improved drug retention and absorption in the nasal cavity, which could translate to enhanced therapeutic outcomes.

### 2.6. Texture Profile Analysis

The desirable attributes of mucoadhesive formulation gels include ease of removal from the primary packaging, ease of application, and the ability to retain the product at the application site without disintegration [61]. These properties are crucial for ensuring effective delivery, prolonged contact with the mucosal surface, and enhanced therapeutic outcomes [61,62]. The texture analysis of the in situ hydrogel loaded with Nanotriphala (Table 3) reveals critical insights into its mechanical and adhesive properties, which directly relate to its mucoadhesive potential for nasal drug delivery [63]. The hardness of the hydrogel, measured at 14.05 ± 0.35 g, indicates its structural stability and ability to withstand external forces during application and retention within the nasal cavity [61]. This value aligns with the findings of Basu et al., who reported that a hardness of less than 28 g is considered optimal for trans-nasal formulations, ensuring both ease of administration and effective retention [64]. This moderate hardness is ideal for nasal formulations, as it ensures the gel is not too rigid to cause discomfort nor too soft to compromise its mechanical integrity during administration. These properties suggest that the formulation can be easily administered in vivo, ensuring smooth and efficient application during use [65].

The recorded adhesiveness was −13.84 ± 1.48 g·s. Our findings show that the adhesive results fall within the range corresponding to those of previous studies [62,66]. In more detail, adhesiveness is related to bioadhesion and is a measure of the force required to overcome the attractive forces between the surfaces of the sample and the probe [67,68]. This property is crucial for ensuring that the formulation can effectively adhere to the mucosal surfaces, allowing for prolonged retention and enhanced therapeutic effects at the application site [67]. The relationship between texture analysis and mucoadhesion is crucial in designing nasal formulations. Mucoadhesion relies on the ability of the hydrogel to adhere to the mucosal surface, which is influenced by its mechanical and adhesive properties [69]. A hydrogel with suitable hardness ensures ease of application without collapsing, while optimal adhesiveness enhances retention within the dynamic environment of the nasal cavity, making it a promising candidate for nasal drug delivery.

### 2.7. Physical Stability of In Situ Hydrogel Loaded with Nanotriphala

**Figure 5:**
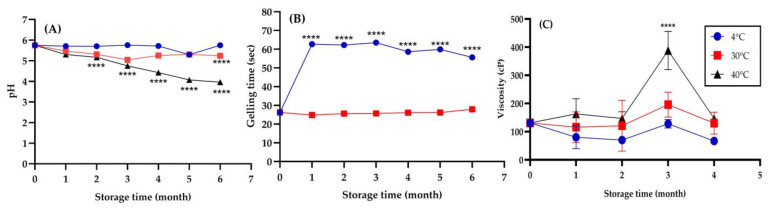
pH stability (**A**), gelling time (**B**), and viscosity (**C**) of In situ Tri at different storage temperatures (4 °C, 30 °C, and 40 °C) over a specified period. The data represent the mean ± standard deviation (SD) of three independent experiments (n = 3). **** indicates a significant difference (*p* < 0.0001) compared to the initial storage period.

In situ hydrogels for nasal drug delivery must be stable during storage to effectively deliver drugs, and temperature and pH are important factors. This study evaluated a thermoresponsive In situ Tri at three storage temperatures (4 °C, 30 °C, and 40 °C) during a six-month stability study. Hydrogel pH was 5.75 ± 0.01 at baseline, an ideal value for nasal mucosa as previously described. However, this showed significant changes over time, where the pH was most stable at 4 °C, with only slight decreases to 5.29 by month 5, and returning to 5.75 by month 6. At 30 °C, the pH value significantly decreased to 5.24 after 6 months of storage compared to the initial value at 0 months. Moreover, the pH level at 40 °C decreased more extensively, with pH decreasing to 3.96 ± 0.01 by month 6 at 40 °C (Figure 5A), indicating accelerated degradation and potential risks for nasal irritation [49]. Gelling time measurements (Figure 5B) revealed similar temperature-dependent behaviors. The initial gelling time of 26.26 ± 1.16 s remained consistent across all temperatures at baseline, which is suitable for nasal delivery. However, at 4 °C, gelling time progressively increased over time, reaching 55.64 ± 0.46 s by month 6, indicating slower gelation. At 30 °C, gelling time remained stable, suggesting it is an optimal storage condition. At 40 °C, premature gelation was observed after incubation, indicating that the formulation at this temperature lacks long-term storage stability. Therefore, data for this temperature could not be presented in Figure 5B. The temperature-sensitive property of the hydrogel was also confirmed by viscosity analysis (Figure 5C). The baseline viscosity of 131 ± 10 cP underwent notable transformations. The viscosity at 4 °C decreased relatively slowly until it was 67 ± 14 cP, whilst that at 30 °C was unstable. At 40 °C the most substantial changes were observed, with the viscosity rocketing up to 388 ± 568 cP by month 3, indicating a degree of nanoparticle aggregation and gelation of the polymer. All these changes can influence mucoadhesion, drug release, and general performance. The viscosity at 4 °C was within an acceptable range for proper nasal application, but at 40 °C, the hydrogel showed high viscosity and impaired effectiveness.

Based on the thorough analysis described above, 4 °C is the most appropriate storage temperature for In situ Tri. This is the condition that best maintains the pH stability, gelling properties, and viscosity of the formulation. For short-term storage, 30 °C seems acceptable, while 40 °C should be avoided because of substantial degradation of critical hydrogel properties. These conclusions highlight the importance of temperature when maintaining the integrity of systems for advanced drug delivery. The temperature sensitivity showcases the fragile aspects of In situ Tri and indicates that appropriate storage conditions must be strictly adhered to in order to maintain optimal therapeutic performance. It is also a significant consideration for storage in the nasal drug delivery system.

### 2.8. Antimicrobial Activities of In Situ Hydrogel Loaded with Nanotriphala

The results of antimicrobial activity for In situ Tri are essential knowledge about their potential activity against bacterial pathogens that are relevant to oxidative stress-related nasal diseases. As shown in Table 4, the minimum inhibitory concentration (MIC), minimum bactericidal concentration (MBC), and MBC/MIC ratios for different microorganisms demonstrate that all the tested strains were susceptible to the formulation’s antimicrobial action. The MBC/MIC ratios of all microorganisms, being less than 4, demonstrate that the concentrations necessary to kill the bacteria (MBC) are near those needed to stop their growth (MIC), thus confirming the bactericidal properties of the formulation [70]. These results align with similar studies where nanostructured formulations demonstrated enhanced antimicrobial efficacy [71]. Overall, In situ Tri has shown excellent broad-spectrum antimicrobial activities against Gram-negative (*E. coli*, *P. aeruginosa*) and Gram-positive (*S. aureus*, *S. epidermidis*) bacteria, indicating that it can provide a new potential antibiotic for oxidative stress-related nasal diseases. The present study also showcased the low minimum inhibitory concentration (MIC) and minimum bactericidal concentration (MBC) values of the examined formulation against *P. aeruginosa*, which indicates the potential of this formulation to target pathogens that have developed resistance to common antibiotic treatment, which is of particular interest as the phenomenon of resistance needs to be investigated more and more in clinical settings. This also reinforces the notion stated here that natural extracts such as Triphala could offer a viable alternative or adjunct to traditional antibiotics [11,72]. The efficacy of the formulation can be attributed to the synergistic actions of bioactive compounds in Triphala, which are further augmented by the nanocarrier system offered by in situ hydrogel, wherein comparable optimization of activity has been reported in other studies utilizing nanoformulations [11]. Furthermore, the enhanced bioavailability and site-specific release of active compounds that can be provided at the nanoscale are important advantages, which were emphasized in recent reviews regarding the involvement of nanomedicine in the treatment of pathogenic microorganisms [73,74]. Additional studies are warranted to explore the specific mechanisms of action, optimal dosing regimens, and therapeutic potential of this formulation for the management of oxidative stress-related bacterial infections in nasal diseases. This highlights the increasing potential of natural product-derived nanotherapeutics as a safe and potent alternative to conventional antibiotic agents against antimicrobial resistance.

### 2.9. Mutagenicity of In Situ Hydrogel Loaded with Nanotriphala

The results of the revertant colony assay for TA98 and TA100 strains with and without S9 activation reveal the mutagenic potential of various treatments (Table 5). The DMSO control, used as a solvent, showed consistent baseline revertant colonies, with values of 31.56 ± 0.56 for TA98 and 28.89 ± 0.48 for TA100 without S9 activation, and 136.89 ± 2.23 and 144.67 ± 1.92 with S9 activation, respectively. For 2AA, a known mutagen, a significant increase in revertant colonies was observed, especially with S9 activation, where the colony count reached 703.11 ± 13.18 for TA98 and 778.67 ± 4.81 for TA100. AF-2, at a concentration of 0.1 µg/plate, showed a moderate mutagenic response, with 349.00 ± 2.22 revertant colonies in TA98 without S9 activation and 786.67 ± 14.05 for TA100 with S9 activation. The blank hydrogel, used as a formulation vehicle, exhibited minimal mutagenic activity, with revertant colonies of 29.78 ± 0.95 for TA98 and 28.22 ± 0.44 for TA100 without S9 activation, and 169.89 ± 1.22 and 179.33 ± 2.85 with S9 activation, respectively. In situ Tri, a formulation containing Nanotriphala, demonstrated no significant mutagenic activity across different concentrations. At 40 µg/plate, the revertant colony count for TA98 was 27.22 ± 0.95 (without S9) and 28.78 ± 1.18 (with S9), and for TA100, it was 134.33 ± 7.43 (without S9) and 145.11 ± 5.10 (with S9). As the concentration increased to 200 µg/plate and 1000 µg/plate, revertant colonies for both strains remained within the range of the control, with no signs of mutagenic effects, indicating that In situ Tri is not mutagenic at these concentrations. At the highest concentration tested, 5000 µg/plate, In situ Tri still showed no significant increase in revertant colonies, with values of 42.22 ± 0.40 for TA98 and 41.56 ± 0.97 for TA100 without S9, and 188.56 ± 2.23 and 202.67 ± 2.36 with S9, respectively. These results suggest that, under the conditions tested, In situ Tri does not exhibit mutagenic potential and could be considered safe in terms of genetic toxicity.

**Figure 6:**
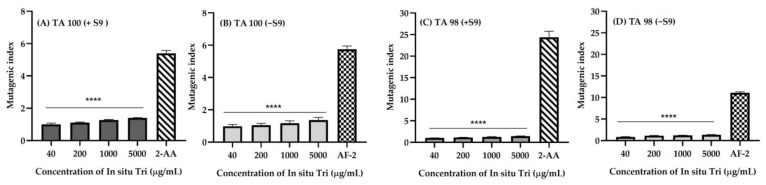
Mutagenic activity expressed by the mean of revertant/plate index of mutagenicity (IM) induced by different concentrations of in situ Tri in the TA98 and TA100 strains of *S. typhimurium* in the presence (+S9) and absence (−S9) of metabolic activation. The data represent the mean ± standard deviation (SD) of three independent experiments (n = 3). **** indicates a significant difference (*p* < 0.0001) when compared to the standard mutant for each condition.

The mutagenicity index, as shown in Figure 6, reveals that for all concentrations of In situ Tri, the mutagenic index is consistently below 2. A mutagenic index equal to or greater than 2 is considered the threshold for classifying a substance as a mutagenic agent [75,76]. These results are in agreement with several mutagenicity studies performed with plant-derived compounds, where nonmutagenicity was also attributed to dose effects [77]. The absence of mutagenic effects in both the presence and absence of the S9-activating enzyme further supports the nonmutagenic nature of In situ Tri, as similar results have been observed in other studies of Triphala extract [78].

For instance, as shown in the following results of the Salmonella/human S9 mutagenicity test, all of the In situ Tri concentrations 40, 200, 1000, and 5000 μg/mL did not affect either viability or metabolic activity of the bacteria/yeast/liver, with readings close to 1.0 indicating that In situ Tri in either non-treated or treated forms is not mutagenic, as mentioned above. Though a slight increase in values with increasing concentrations might indicate a possible growth-stimulating effect, these values still fell within an expected range for nonmutagenicity. Mutagen control AF-2 displayed much higher numbers of revertant colonies, especially in the TA 98 strains, confirming its status as a highly mutagenic constituent of the plant. The increased sensitivity of TA 98 strains, in comparison to the TA 100 strain responsive to base-pair substitutions, to frame-shift mutations is consistent with the specificity of AF-2’s mutagenic effects [79].

Based on the mutagenicity test results, In situ Tri does not exhibit any detectable mutagenic activity. These findings support a positive safety profile for the In situ Tri formulation, particularly for therapeutic use in addressing oxidative stress-related nasal disorders. Additionally, there is evidence suggesting that Triphala-based products are nonmutagenic, which is consistent with the results of this mutagenicity test [78]. This further reinforces the safety and suitability of In situ Tri for potential clinical applications.

### 2.10. Antimutagenic Activity of In Situ Hydrogel Loaded with Nanotriphala

**Figure 7:**
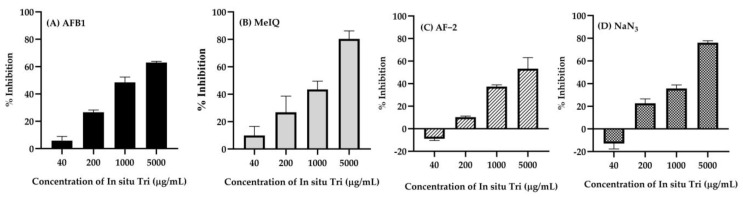
Antimutagenicity of various concentrations of in situ *Tri* against the mutagenicity of indirect mutagens, (**A**) AFB1 in TA98 and (**B**) MeIQ in TA100, as well as direct mutagens: (**C**) AF−2 in TA98 and (**D**) NaN_3_ in TA100. Values are expressed as mean ± SD (n = 3).

The antimutagenic activity was assessed by comparing the number of revertant colonies of the test substances with those of the mutagen alone, as shown in Figure 7A–D. The inhibition percentage (% inhibition) of mutagenicity was calculated with the Ames test, indicating a moderate antimutagenic effect when the inhibitory effect is between 25 and 40% and a strong effect when it exceeds 45%. Inhibitory effects of less than 25% are considered weak [80]. The antimutagenic activity of In situ *Tri* was assessed by evaluating its inhibitory effects against various mutagens, including the indirect mutagen Aflatoxin B1 (AFB1) in the TA98 strain, MeIQ in the TA100 strain, and the direct mutagens AF-2 in the TA98 strain and sodium azide (NaN3) in the TA100 strain. At a concentration of 40 µg/mL, In situ *Tri* exhibited weak inhibition across the tested mutagens. The inhibition against AFB1 was 5.91 ± 3.01%, while AF-2 showed a negative inhibition of −8.81 ± 1.59%, suggesting a potential enhancing effect rather than inhibition. MeIQ showed a modest 9.91 ± 5.59% inhibition, and NaN_3_ inhibition was negative at −12.87 ± 4.74%, indicating little to no antimutagenic activity at this low concentration. At 200 µg/mL, moderate inhibition was observed for most mutagens. AFB1 exhibited 26.6 ± 1.65% inhibition, AF-2 showed 10.20 ± 1.06%, MeIQ had 26.83 ± 11.81%, and NaN_3_ exhibited 22.64 ± 3.95% inhibition. These results suggest a moderate antimutagenic effect, particularly for AFB1 and MeIQ. At 1000 µg/mL, In situ *Tri* demonstrated stronger inhibition, with AFB1 showing 48.47 ± 3.91%, AF-2 showing 37.46 ± 1.14%, MeIQ showing 43.46 ± 6.12%, and NaN_3_ showing 35.73 ± 3.10%. These results indicate moderate to strong antimutagenic activity across the mutagens tested. At the highest concentration of 5000 µg/mL, In situ Tri demonstrated strong inhibitory effects across all mutagens, with AFB1 showing 62.95 ± 0.96%, AF-2 at 53.34 ± 9.79%, MeIQ at 80.41 ± 5.77%, and NaN_3_ at 76.12 ± 1.74%. Our findings align with the previous study by Kaur et al., which reported significant antimutagenic activity of Triphala in the TA98 and TA100 bacterial strains [78]. In more detail, gallic acid, a major component of Triphala, has been suggested in previous reports to exhibit antimutagenic activity through several mechanisms, one of which could involve gallic acid acting as a nucleophile to scavenge electrophilic mutagens [81]. These results indicate that In situ Tri exhibits potent antimutagenic activity, particularly against MeIQ and NaN_3_, at higher concentrations, providing strong evidence of its potential as an effective antimutagenic agent.

## 3. Conclusions

Overall, this wide-ranging investigation of the thermoresponsive in situ hydrogel loaded with Nanotriphala sheds light on its role as an innovative and potential nasal drug delivery system in the treatment of oxidative stress-induced nasal disorders. The Nanotriphala formulation exhibited excellent antioxidant activity in more than one assay and its radical-scavenging potential was severalfold greater than that of the existing Triphala extract. The nanoencapsulation method employed in this study significantly enhanced the biological efficacy of active components, as evidenced by a marked reduction in IC_50_ values in DPPH, ABTS, and FRAP assays compared to Triphala extract, effectively demonstrating its superior ability to alleviate oxidative stress. Antimicrobial studies showed broad-spectrum activity against several Gram-positive and Gram-negative strains of bacteria, with improved efficacy against difficult pathogens such as *Pseudomonas aeruginosa*. The formulation’s improved antimicrobial properties suggest its potential therapeutic benefit in complicated nasal infections of oxidative stress origin. The anti-inflammatory property of the Nanotriphala was assessed by the nitric oxide inhibition assays, which showed concentration-dependent effects, highlighting its potential therapeutic applications in managing oxidative stress-related inflammatory diseases. After incorporating Nanotriphala into an in situ hydrogel system, the resulting formulation exhibited excellent physicochemical characteristics, positioning it as a promising therapeutic strategy. With a physiological pH of 5.75 ± 0.01, a gelling temperature of 34.0 °C, and a rapid gelation time of 26.27 ± 1.16 s, the hydrogel demonstrated optimal properties for nasal application. It also displayed good spreadability (10.25 ± 0.28 cm) and suitable hardness and adhesiveness, ensuring enhanced nasal drug delivery performance. These features promote prolonged contact with the mucosa, minimize clearance, and enable engineered drug-release profiles, addressing significant challenges faced by nasal therapies in clinical practice. The biocompatibility and safety profile of Nanotriphala hydrogel at different concentrations were confirmed by high cell viability and its reverse mutagenic potential in Salmonella typhimurium strains. Stability studies strongly recommended achieving ideal physicochemical properties of the formulation at a storage temperature of 4 °C. The temperature sensitivity demonstrates the delicate nature of in situ hydrogel loaded with Nanotriphala, highlighting the importance of exact storage conditions to preserve therapeutic efficacy.

In summary, the thermoresponsive in situ hydrogel loaded with Nanotriphala has demonstrated significant potential as a novel nasal delivery system for managing nasal disorders. Our findings highlight its optimal properties for nasal application, including an excellent safety profile, along with remarkable anti-inflammatory, antibacterial, antioxidant, and antimutagenic activities. This research provides strong preliminary evidence supporting the use of Nanotriphala-loaded in situ hydrogel as an effective therapeutic approach for addressing complex nasal disorders.

## 4. Materials and Methods

Poloxamer 407 was sourced from Chanjao Longevity Co., Ltd. (Bangkok, Thailand), and polyethylene glycol 400 was obtained from Dow Corporate Headquarters (Midland, MI, USA). *Staphylococcus aureus* DMST 8013, *Pseudomonas aeruginosa* DMST 15501 and *Candida albicans* DMST 5815 were purchased from Department of Medical Sciences, Ministry of Public Health, Thailand (DMST). *Aspergillus niger* ATCC 10578 was purchased from American Type Culture Collection, Manassas, VA, USA. The cetrimide agar base was from Himedia, Mumbai, Maharashtra, India. Glycerol was purchased from SL quality supply, Bangkok, Thailand. Standard gallic acid with 99.6% purity was purchased from Sigma-Aldrich, USA. HPLC grades of acetonitrile and methanol were obtained from J.T. Baker, Phillipsburg, NJ, USA. HPLC water was purchased from RCI lab-scan, Bangkok Thailand. Potassium dihydrogen phosphate, AR grade, was obtained from Merck, Darmstadt, Hesse, Germany.

### 4.1. Preparation of Nanotriphala

Triphala nanoparticles were formulated based on our previous method [23]. Briefly, 500 mg of Triphala ethanolic extract was dissolved in 1 mL of acetone and added dropwise at 1 mL per hour to a solution containing 8 mL deionized water, 1 mL of 1% (*w*/*v*) poloxamer 407, and 1 mL of PEG 400, under continuous magnetic stirring at 600 rpm. The mixture was stirred overnight to allow complete evaporation of the organic solvent. For the blank control, the organic solvent was added to the surfactant and stabilizer solution without Triphala extract, leading to micelle formation when the PEO-PPO block copolymer concentration reached its critical micellization concentration (CMC). The nanoparticle size, polydispersity index (PDI), and zeta potential of the Nanotriphala (5 mg/mL) were analyzed using dynamic light scattering (DLS) at a 173° scattering angle and 25 °C with a Zetasizer Nanoseries (Malvern Instruments, Malvern, UK).

### 4.2. Antioxidant Activities of Nanotriphala

#### 4.2.1. DPPH Free Radical-Scavenging Activity Assay

The free radical-scavenging capacity of both Triphala extract (Tri ext) and Nanotriphala (Tri Np) was evaluated using the DPPH assay, following the method described by Chittasupho et al. [82]. Gallic acid was used as the positive control, prepared in concentrations ranging from 0.4 to 200 μg/mL. Aliquots of Triphala extract (1.95 to 1000 μg/mL) and Nanotriphala (0.1 to 50 mg/mL) were prepared. The concentrations of Nanotriphala (Tri Np) used in the DPPH, ABTS, and FRAP experiments were based on the weight of the Nanotriphala formulation (0.1–50 mg/mL). This weight corresponds to an equivalent weight of Triphala extract (Tri ext) ranging from 4.43 to 2000 µg/mL, aligning with the concentrations used for Triphala extract in the same assays. For the assay, 100 μL of each sample was added to a 96-well plate. Following this, 100 μL of a 500 µM DPPH solution was added to each well. The mixture was incubated in the dark for 30 min, after which absorbance was measured at 517 nm. The percentage of inhibition for both the standard and the extract was calculated at each concentration, and data were plotted as % inhibition versus log concentration. The IC_50_ values, representing the concentration needed to reduce DPPH absorbance by 50% compared to the control, were determined for each sample.

DPPH Free radical scavenging (%) = [(A − B)/A] × 100

where A is the absorbance of the reaction with solvent control, and B is the absorbance of the DPPH with the extract or Nanotriphala.

#### 4.2.2. ABTS Free Radical-Scavenging Activity Assay

The ABTS radical-scavenging assay was conducted to further evaluate the free radical-scavenging activity of Tri ext and Tri Np, following the method described by Chittasupho et al. [82]. The ABTS radical stock solution was prepared by mixing 7 mM ABTS solution with 2.45 mM potassium persulfate in a 1:1 ratio and allowing the mixture to react in the dark at room temperature for 12 h. For the assay, 20 μL of gallic acid (0.4 to 200 µg/mL), Triphala extract (1.95 to 1000 µg/mL), and Nanotriphala (0.1 to 50 mg/mL) were added to a 96-well plate, followed by the addition of 180 μL of the ABTS solution. The plates were incubated in the dark for 30 min, after which the absorbance was measured at 734 nm. The percentage of inhibition was calculated for both the standard and the extract, and the IC_50_ values were determined from the resulting graph.

ABTS Free radical scavenging (%) = [(A − B)/A] × 100

where A is the absorbance of the reaction with solvent control, and B is the absorbance of the reaction with the extract.

#### 4.2.3. Ferric-Reducing Antioxidant Power (FRAP) Assay

The FRAP assay was conducted following the method described by Chittasupho et al. [82]. The FRAP reagent was prepared by combining 300 mM acetate buffer (pH 3.6), 10 mM 2,4,6-tripyridyl-s-triazine (TPTZ) solution in 40 mM HCl, and 20 mM FeCl_3_·6H_2_O solution in a 10:1:1 ratio. Gallic acid (0.4 to 200 µg/mL) served as the standard, while solutions of Triphala extract (1.95 to 1000 µg/mL) and Nanotriphala (0.1 to 50 mg/mL) were also prepared. Samples (20 μL) were incubated with 180 μL of FRAP solution in 96-well plates at 37 °C for 30 min in the dark. The absorbance of the ferrous tripyridyltriazine complex was measured at 595 nm. The FRAP content of the extract was expressed as μM Fe (II) equivalent, calculated using a standard curve constructed with ferrous sulfate solution (9.8–5000 μM).

### 4.3. Antimicrobial Activity of Triphala Extract and Nanotriphala

The antimicrobial activities of Triphala extract and Nanotriphala were evaluated using the broth dilution technique. Minimum inhibitory concentrations (MICs) and minimum bactericidal concentrations (MBCs) against *Escherichia coli* (ATCC 25922), *Pseudomonas aeruginosa* (DMST15501), *Staphylococcus aureus* (DMST8013), and *Staphylococcus epidermidis* (DMST15055) were determined by the broth microdilution method [83]. Triphala extract was tested at concentrations of 32–1024 µg/mL, and Nanotriphala at 390–25,000 µg/mL. The MIC was defined as the lowest concentration that completely inhibited bacterial growth. MBC values were determined by the drop plate technique and recorded as the lowest concentration, showing no bacterial growth on TSA plates.

### 4.4. Cell Culture

The RAW264.7 (ATCC TIB-71) cell line was obtained from Dr. Natthachai Duangnin (Regional Medical Science Center 1, Chiang Mai, Thailand). Cells were cultured in Dulbecco’s Modified Eagle’s Medium (DMEM) with high glucose, supplemented with 10% fetal bovine serum (FBS) and 1% penicillin–streptomycin, and incubated at 37 °C in 5% CO_2_. Subculturing was performed every three days using 0.25% trypsin-EDTA.

### 4.5. Cytotoxicity Assay of Triphala Extract and Nanotriphala Against RAW264.7 Cells

The cytotoxicity of gallic acid, Triphala extract, and Nanotriphala against RAW264.7 cells was evaluated to determine the maximum concentration suitable for use in the NO assay, an anti-inflammatory test. Cell viability was assessed at various concentrations of gallic acid, Triphala extract, and Nanotriphala using the MTT assay, following the method described by Chittasupho et al. [84]. RAW264.7 cells were seeded in 96-well plates at a density of 8 × 10^3^ cells/well in culture medium and incubated at 37 °C with 5% CO_2_. After a 24 h adhesion period, Triphala extracts and Nanotriphala were applied to the cells, followed by incubation at 37 °C in 5% CO_2_ for 24 h. At 24 h post-treatment, an MTT solution (0.5 mg/mL in culture medium) was added to each well, and the cells were incubated for an additional 2 h at 37 °C. Formazan crystals formed by metabolically active cells were dissolved in 100 µL DMSO per well, and absorbance was measured at 550 nm. IC_50_ values were calculated using GraphPad Prism v.7.0 (La Jolla, CA, USA).

### 4.6. Anti-Inflammation Activity Assay of Triphala Extract and Nanotriphala

The nitric oxide (NO) assay was employed to assess the anti-inflammatory activity of the Triphala extract and Nanotriphala by measuring nitrite, the final stable product of nitric oxide in the culture medium. Nitric oxide secretion was evaluated in RAW 264.7 macrophages following the method described by Chittasupho et al. [85]. RAW 264.7 macrophage cells were seeded at a density of 1 × 10^5^ cells per well in 96-well plates and incubated for 24 h. Following this, the cells were pretreated with Triphala extract (15.6–2000 µg/mL), Nanotriphala (62.5–8000 µg/mL), or gallic acid standard (1.95–250 µg/mL) for 1 h. After pretreatment, cells were exposed to LPS (50 ng/mL) to induce an inflammatory response. After 18 h of incubation, nitric oxide levels in the supernatant were measured using the Griess reagent following the previous report [85]. The supernatant from each well was collected to assess nitrite levels, with absorbance measured at 540 nm. Nitrite concentrations were quantified based on a standard nitrite calibration curve. Results were expressed as the percentage (%) of nitrite secreted by treated cells relative to the control (LPS-treated cells).

### 4.7. Preparation of In Situ Hydrogel Loaded with Nanotriphala

The in situ hydrogel containing Nanotriphala (In situ Tri) was prepared by dissolving Nanotriphala (1 g), 0.25% *w*/*v* benzalkonium chloride (4 g), and sodium chloride (0.76 g) in deionized water (77.8 g). The above excipients were stirred thoroughly using an overhead stirrer set to 600 rpm until a homogenous solution was achieved. Next, poloxamer 188 was slowly added to the solution by sprinkling it gradually while maintaining continuous stirring until fully dissolved. Then, poloxamer 407 and introduced into the solution and stirred continuously for another 2 h. The clear solution was stored at 4 °C for 24 h and was transferred into a spray bottle for subsequent application.

### 4.8. Characterization of In Situ Hydrogel Loaded with Nanotriphala

#### 4.8.1. Gelation Temperature

The gelation temperature is defined as the temperature at which the drug solution transforms into a gel. This was determined using the test tube inversion method [86]. A 10 mL amount of In situ Tri was placed in a test tube within a water bath. The solution was then heated. A thermometer was immersed in the solution to monitor temperature continuously. The gelation temperature was recorded as the point at which the solution ceased movement due to gel formation. This experiment was performed in triplicate.

#### 4.8.2. Gelation Time

The gelling time of the In situ Tri formulation was assessed using the test tube inversion method described by Sherafudeen et al. [86]. Specifically, 10 mL of the formulation was added to a glass test tube. The test tube was then placed in a temperature-controlled water bath set at 34 °C. Periodically, the test tube was inverted at a 90° angle, and the time required for the liquid to gel was recorded in seconds as the gelation time.

#### 4.8.3. pH

The pH of the developed In situ Tri formulation was measured using a calibrated digital pH meter (Mettler Toledo, OH, USA). Prior to measurement, the pH meter was calibrated with standard buffer solutions. The pH was determined by immersing the electrode fully into the formulation at room temperature. Measurements were conducted in triplicate, and the results are reported as the average value.

#### 4.8.4. Rheology and Viscosity

The amount of gel that can be introduced into the nasal cavity is limited due to its small volume. Therefore, the rheology and viscosity of the In situ Tri must be low at the time of application to facilitate easy delivery but should increase afterward to ensure the drug remains at the disease site for a sufficient duration. The viscosity of In situ Tri formulations was measured using Haake Modular Advance Rheometer System MARS 40 Rheometer (Thermo Fisher Scientific Inc., MA, USA). The rheology and viscosity of formulation were evaluated at 25 °C.

#### 4.8.5. Ex Vivo Spreadability

The spreadability was evaluated based on the method described by Sherafudeen et al. with slight modifications [86]. A 10 × 4 cm rectangular glass slide was used to assess spreadability. The porcine nasal mucosa, with the serosal side facing outward, was secured onto the slide using a thread. The slide was then placed in a hot air oven at 37 °C. A single drop of In situ Tri was applied to the mucosa at a 120° angle. Spreadability was determined by measuring the distance traveled by the In situ Tri drop before it gelled. The average of three measurements was recorded. The protocol for the use of cadavers was approved by the Animal Care and Use Committee, Faculty of Veterinary Medicine, Chiang Mai University, Thailand (FVM-CMU-ICUC Ref. No. 2567/BR-0001. C2).

#### 4.8.6. Quantification of Gallic Acid in In Situ Hydrogel Loaded with Nanotriphala Formulation by High-Performance Liquid Chromatography (HPLC)

Chromatographic separation was performed using a Shimadzu LC-10ATvp high-performance liquid chromatography (HPLC) system equipped with a UV detector (Shimadzu, Kyoto, Japan). Separation was carried out on a Mightysil C18 column (250 mm × 4.6 mm, 5.0 µm particle size; Kanto Chemical Co., Inc., Tokyo, Japan) at a column temperature of 17 °C. A 20 μL aliquot of the sample was injected, and detection was conducted at 270 nm. The flow rate was set to 1.0 mL/min. Gradient elution was employed using a mobile phase consisting of solvent A (acetonitrile) and solvent B (20 mM potassium dihydrogen phosphate buffer), with the following gradient: 1:90 (A) at 0 min, 1:90 at 5 min, 70:30 at 20 min, 10:90 at 25 min, and 10:90 at 35 min. For calibration, a standard curve was generated using gallic acid standard solutions at five concentrations ranging from 3.001 to 9.004 µg/mL. The calibration curve was constructed by plotting peak areas against the corresponding concentrations, with triplicate analysis of the gallic acid standard.

Sample preparation involved dissolving 500 mg of the In situ Tri formulation in 0.5 mL methanol, followed by shaking at 2500 rpm and sonication for 10 min at temperatures below 30 °C. The mixture was then centrifuged at 10,900 rpm for 10 min and filtered through a 0.2 µm PTFE syringe filter. Prior to analysis, 450 µL of the supernatant was mixed with 50 µL of 20 mM potassium dihydrogen phosphate buffer, shaken, and transferred to an injection vial for HPLC analysis

### 4.9. Texture Profile Analysis of In Situ Hydrogel Loaded with Nanotriphala

Texture profile analysis was conducted following the method described by Xu et al., with slight modifications [87]. The texture parameters, including hardness, stickiness, and adhesiveness, were measured using a TA-XT Plus texture analyzer (Stable Micro System, Surrey, UK) in a simple compression mode. For the experiment, 10 g of the sample was transferred into a 15 mL glass vial to achieve a sample height of 25 mm. The beaker was kept in a thermostatic water bath at a temperature of 34 ± 1 °C. A spherical analytical probe (Type P/0.5HS) was used, which was lowered at a speed of 2 mm/s to a depth of 10 mm before being retracted at a faster rate of 10 mm/s until it fully detached from the gel surface. The resulting force–distance curve was analyzed using Texture Exponent 32 software to determine hardness, stickiness, and adhesiveness. All tests were performed in triplicate.

### 4.10. Physical Stability of In Situ Hydrogel Loaded with Nanotriphala

To assess the stability of the In situ Tri formulation, samples were stored at 4 °C, 30 °C, and 40 °C for three months, with gelation time, gelation temperature, pH, rheological properties, and viscosity measured at baseline (0 months), 1 month, 2 months, and 3 months. Aliquots were placed in individual containers and monitored at each time point to observe potential changes due to temperature. The physical characteristics of samples were determined according to the above methods.

### 4.11. Antimicrobial and Microbiological Stability of In Situ Hydrogel Loaded with Nanotriphala

The microbial limitation assay was assessed by measuring total aerobic microbial counts (TAMCs) and total yeast and mold counts (TYMCs). This was performed using 3M^TM^ Petrifilm^TM^ aerobic count plates and 3M^TM^ Petrifilm^TM^ yeast and mold count plates. Positive controls for TAMC included *S. aureus* (DMST 8013) and *P. aeruginosa* (DMST 15501), while positive controls for TYMC were *Candida albicans* (DMST 5815) and *Aspergillus niger* (ATCC 10578). The 3M^TM^ Petrifilm^TM^ Staph Express plate and Cetrimide agar plate were used to examine the contamination of specified microorganisms, *S. aureus* and *P. aeruginosa*, respectively. The positive control for the presence of *S. aureus* on 3M^TM^ Petrifilm^TM^ Staph Express plate was *S. aureus*, and the positive control for the presence of *P. aeruginosa* on the Cetrmide agar plate was *P. aeruginosa*. In situ Tri contamination was identified according to standards set by the U.S. Pharmacopoeia (USP) 41, British Pharmacopoeia (BP) 2024, and Thai Herbal Pharmacopoeia 2022.

### 4.12. Mutagenicity Determination of In Situ Hydrogel Loaded with Nanotriphala

The mutagenicity of Triphala extract and In situ Tri was assessed using the Ames test, following the method described by Punvittayagul et al. [88]. The assay utilized *Salmonella typhimurium* strains TA98 and TA100, with and without an exogenous metabolic activation system (S9 mix). Distilled water or DMSO served as the negative control, and the standard mutagens 2-AA and AF-2 were used as positive controls in the presence of metabolic activation and the presence of metabolic activation, respectively.

The number of mutant bacterial colonies was counted after incubating the test substance (or standard mutagen) with the bacteria and liver enzyme (or buffer in the absence of enzyme activation) for 48 h on nutrient agar. The mutagenic index was then calculated (number of revertant colonies of the test substance divided by the number of revertant colonies in the negative control). If the mutagenic index was greater than 2, the test sample was considered to have potential mutagenic effects.

### 4.13. Antimutagenicity Test of In Situ Hydrogel Loaded with Nanotriphala

For the antimutagenicity test, the assay steps were conducted following the same procedure as the mutagenicity test, as described in the method of Punvittayagul et al. [88]. In the study of the antimutagenic effects of the test substance, the mutagens used to induce bacterial mutation were divided into two groups. The first group consists of indirect mutagens, which require the activity of enzymes to alter their structure. The mutagens used in this study included Aflatoxin B1 (AFB1), which was tested in TA98, and 3-methyl-3, 4-dimethyl-3H-imidazo[4,5-f]quinolin-2-amine (MeIQ), which was tested in TA100. The second group is direct mutagens, which do not require enzyme activation. The direct mutagens used in this study were 2-(2-furyl)-3-(5-nitro-2-furyl)-acrylamide (AF-2), tested in the TA98 strain, and sodium azide (NaN_3_), tested in the TA100 strain. The number of histidine-independent revertant colonies was counted after incubation and compared with the positive control. Antimutagenicity was expressed as the percentage of inhibition of mutagenicity using the following formula:

% Inhibition = {[(A − B) − (C − B)]/(A − B)} × 100

where A represents the number of revertants in standard mutagen plates, B denotes the number of spontaneous revertants, and C corresponds to the number of revertants observed in the test plates.

### 4.14. Statical Analysis

All experiments were performed in triplicate. All data are presented as mean ± standard deviation (mean ± S.D.) values. Prism version 8.0 software was used for statistical analysis using an independent *t*-test, one-way ANOVA, and two-way ANOVA. Statistical significance was determined at * *p* < 0.05 and **** *p* < 0.0001.

## Figures and Tables

**Table 1:** MIC and MBC of Triphala extract and Nanotriphala for different microorganism strains.

Sample	MIC:MBC (µg/mL)			
	*E. coli*	*P. aeruginosa*	*S. aureus*	*S. epidermidis*
Tri ext	128:1024	>1024:>1024	1024:>1024	1024:>1024
Tri Np	390:1562	12,500:25,000	1562:12,500	1562:6250
Antibiotics	0.5:0.5 ^Gen^	0.5:1 ^Gen^	0.5: 0.5 ^VA^	0.5:1 ^VA^

Gen: Gentamicin; VA: Vancomycin. The data represent the mean of three independent experiments (n = 3).

**Table 2:** Characterization of in situ gel.

Sample	pH	Gelation Time (s)	Gelation Temperature (°C)	Viscosity (cP)	Spreadability (cm)	Gallic Acid Content (µg/100 mL)
In situ Tri	5.75 ±0.01	26.27 ± 1.16	34.0 ± 0.0	227 ± 22	10.25 ± 0.28	0.5796 ± 0.0218

Values are mean ± SD. The data represent the mean of three independent experiments (n = 3).

**Table 3:** Texture profile of in situ hydrogel loaded with Nanotriphala.

	Hardness (g)	Adhesiveness (g·s)
In situ Tri	14.05 ± 0.35	−13.84 ± 1.48

Values are mean ± SD. The data represent the mean of three independent experiments (n = 3).

**Table 4:** MIC and MBC of in situ hydrogel loaded with Nanotriphala for different microorganism strains.

Microorganism	µg/mL			
	MIC	MBC	MBC/MIC	Activity
*E. coli*	128	32	<4	Bactericidal
*P. aeruginosa*	4	2	<4	Bactericidal
*S. aureus*	32	4	<4	Bactericidal
*S. epidermidis*	32	8	<4	Bactericidal

The data represent the mean of three independent experiments (n = 3).

**Table 5:** Mutagenicity of in situ hydrogel loaded with Nanotriphala (In situ Tri).

Treatment	Concentration (µg/Plate)	Average of Revertant Colonies			
		TA 98		TA 100	
		−S9	+S9	−S9	+S9
DMSO	50 (µL/plate)	31.56 ± 0.56	28.89 ± 0.48	136.89 ± 2.23	144.67 ± 1.92
2AA	0.5	N/A	703.11 ± 13.18	N/A	778.67 ± 4.81
AF-2	0.1	349.00 ± 2.22	N/A	786.67 ± 14.05	N/A
Blank hydrogel	5000	29.78 ± 0.95	28.22 ± 0.44	169.89 ± 1.22	179.33 ± 2.85
In situ Tri	40	27.22 ± 0.95	28.78 ± 1.18	134.33 ± 7.43	145.11 ± 5.10
	200	36.44 ± 0.97	32.89 ± 0.99	143.00 ± 8.19	160.56 ± 1.25
	1000	38.11 ± 0.56	36.67 ± 1.07	161.22 ± 9.88	182.22 ± 1.46
	5000	42.22 ± 0.40	41.56 ± 0.97	188.56 ± 2.23	202.67 ± 2.36

Values are mean ± SD. AF-2: 2-(2-furyl)-3-(5-nitro-2-furyl)-acrylamide, 2-AA: 2-amino anthracine, NA: Not analyzed. The data represent the mean of three independent experiments (n = 3).

## Data Availability

The data that support the findings of this study are available from the corresponding author upon reasonable request.

## References

[1] Sies H., Berndt C., Jones D.P. (2017). Oxidative stress. Annu. Rev. Biochem..

[2] Tai J., Shin J.-M., Park J., Han M., Kim T.H. (2023). Oxidative stress and antioxidants in chronic rhinosinusitis with nasal polyps. Antioxidants.

[3] Holguin F. (2013). Oxidative stress in airway diseases. Ann. Am. Thorac. Soc..

[4] Kahveci O.K., Kuzu S., Altıntaş M., Vurmaz A., Çelik S. (2021). The effect of nasal steroid and antihistamine use on total oxidative stress and antioxidant status in the treatment of allergic rhinitis. Am. J. Rhinol. Allergy.

[5] Arnaudova Danevska I., Jakjovska T., Atanasovska E., Petrushevska M., Dzekova Vidimliski P., Krstevska Balkanov S., Balkanov T., Zendelovska D. (2023). Effect of nasal corticosteriods on oxidative stress parameters in children with allergic rhinitis. J. Morphol. Sci..

[6] Tai J., Lee K., Kim T.H. (2021). Current perspective on nasal delivery systems for chronic rhinosinusitis. Pharmaceutics.

[7] Djupesland P.G. (2013). Nasal drug delivery devices: Characteristics and performance in a clinical perspective—A review. Drug Deliv. Transl. Res..

[8] Charoenchai L., Pathompak P., Madaka F., Settharaksa S., Saingam W. (2016). HPLC-MS profiles and quantitative analysis of triphala formulation. Interprof. J. Health Sci..

[9] Nariya M., Shukla V., Jain S., Ravishankar B. (2009). Comparison of enteroprotective efficacy of triphala formulations (Indian Herbal Drug) on methotrexate-induced small intestinal damage in rats. Phytother. Res. Int. J. Devoted Pharmacol. Toxicol. Eval. Nat. Prod. Deriv..

[10] Prasad S., Srivastava S.K. (2020). Oxidative stress and cancer: Chemopreventive and therapeutic role of triphala. Antioxidants.

[11] Omran Z., Bader A., Porta A., Vandamme T., Anton N., Alehaideb Z., El-Said H., Faidah H., Essa A., Vassallo A. (2020). Evaluation of antimicrobial activity of Triphala constituents and nanoformulation. Evid.-Based Complement. Altern. Med..

[12] Kalaiselvan S., Rasool M.K. (2016). Triphala herbal extract suppresses inflammatory responses in LPS-stimulated RAW 264.7 macrophages and adjuvant-induced arthritic rats via inhibition of NF-κB pathway. J. Immunotoxicol..

[13] Jantrapirom S., Hirunsatitpron P., Potikanond S., Nimlamool W., Hanprasertpong N. (2021). Pharmacological benefits of Triphala: A perspective for allergic rhinitis. Front. Pharmacol..

[14] Huang H.-Z., Zhao S.-Y., Ke X.-M., Lin J.-Z., Huang S.-S., Xu R.-C., Ma H.-Y., Zhang Y., Han L., Zhang D.-K. (2018). Study on the stability control strategy of Triphala solution based on the balance of physical stability and chemical stabilities. J. Pharm. Biomed. Anal..

[15] Huang L., Huang X.-H., Yang X., Hu J.-Q., Zhu Y.-Z., Yan P.-Y., Xie Y. (2024). Novel nano-drug delivery system for natural products and their application. Pharmacol. Res..

[16] Wang X., Liu G., Ma J., Guo S., Gao L., Jia Y., Li X., Zhang Q. (2013). In situ gel-forming system: An attractive alternative for nasal drug delivery. Crit. Rev. Ther. Drug Carr. Syst..

[17] Nazar H., Fatouros D., van der Merwe S., Bouropoulos N., Avgouropoulos G., Tsibouklis J., Roldo M. (2011). Thermosensitive hydrogels for nasal drug delivery: The formulation and characterisation of systems based on N-trimethyl chitosan chloride. Eur. J. Pharm. Biopharm..

[18] Karnaki A., Siamidi A., Karalis V., Lagopati N., Pippa N., Vlachou M. (2024). Thermoresponsive Hydrogels: Current Status and Future Perspectives. Bioinspired Technology and Biomechanics.

[19] Kumar R. (2023). Nanotechnology in herbal medicine: Challenges and future perspectives. Nanotechnology in Herbal Medicine.

[20] Moradi S.Z., Momtaz S., Bayrami Z., Farzaei M.H., Abdollahi M. (2020). Nanoformulations of herbal extracts in treatment of neurodegenerative disorders. Front. Bioeng. Biotechnol..

[21] Namuga C., Ocan M., Kinengyere A.A., Richard S., Namisango E., Muwonge H., Kirabira J.B., Lawrence M., Obuku E.A. (2023). Efficacy of nano encapsulated herbal extracts in the treatment of induced wounds in animal models: A systematic review protocol. Syst. Rev..

[22] Horn R.C., Vargas V.M.F. (2003). Antimutagenic activity of extracts of natural substances in the Salmonella/microsome assay. Mutagenesis.

[23] Chittasupho C., Umsumarng S., Srisawad K., Arjsri P., Phongpradist R., Samee W., Tingya W., Ampasavate C., Dejkriengkraikul P. (2024). Inhibition of SARS-CoV-2-Induced NLRP3 Inflammasome-Mediated Lung Cell Inflammation by Triphala-Loaded Nanoparticle Targeting Spike Glycoprotein S1. Pharmaceutics.

[24] Lofts A., Campea M.A., Winterhelt E., Rigg N., Rivera N.P., Macdonald C., Frey B.N., Mishra R.K., Hoare T. (2024). In situ-gelling hydrophobized starch nanoparticle-based nanoparticle network hydrogels for the effective delivery of intranasal olanzapine to treat brain disorders. Int. J. Biol. Macromol..

[25] Abdullah J.A.A., Perdomo C.A.A., Núnez L.A.H., Rivera-Flores O., Sánchez-Barahona M., Guerrero A., Romero A. (2024). Lychee peel extract-based magnetic iron oxide nanoparticles: Sustainable synthesis, multifaceted antioxidant system, and prowess in eco-friendly food preservation. Food Bioprod. Process..

[26] Niedzielska E., Smaga I., Gawlik M., Moniczewski A., Stankowicz P., Pera J., Filip M. (2016). Oxidative stress in neurodegenerative diseases. Mol. Neurobiol..

[27] Muscolo A., Mariateresa O., Giulio T., Mariateresa R. (2024). Oxidative stress: The role of antioxidant phytochemicals in the prevention and treatment of diseases. Int. J. Mol. Sci..

[28] Fisher D.R., Zheng T., Bielinski D.F., Kelly M.E., Cahoon D.S., Shukitt-Hale B. (2022). Phytochemical combination is more effective than individual components in reducing stress signaling in rat hippocampal neurons and microglia in vitro. Int. J. Mol. Sci..

[29] Zhang L., Virgous C., Si H. (2019). Synergistic anti-inflammatory effects and mechanisms of combined phytochemicals. J. Nutr. Biochem..

[30] Ling J.K.U., Sam J.H., Jeevanandam J., Chan Y.S., Nandong J. (2022). Thermal degradation of antioxidant compounds: Effects of parameters, thermal degradation kinetics, and formulation strategies. Food Bioprocess Technol..

[31] Zargoosh Z., Ghavam M., Bacchetta G., Tavili A. (2019). Effects of ecological factors on the antioxidant potential and total phenol content of Scrophularia striata Boiss. Sci. Rep..

[32] Muteeb G. (2023). Nanotechnology—A light of hope for combating antibiotic resistance. Microorganisms.

[33] Culas M., Popovich D., Rashidinejad A. (2023). Recent advances in encapsulation techniques for cinnamon bioactive compounds: A review on stability, effectiveness, and potential applications. Food Biosci..

[34] Bhattacharya A., Ghosal S., Bhattacharya S. (2000). Antioxidant activity of tannoid principles of *Emblica officinalis* (amla) in chronic stress induced changes in rat brain. Indian J. Exp. Biol..

[35] Mishra S., Anuradha J., Tripathi S., Kumar S. (2016). In vitro antioxidant and antimicrobial efficacy of Triphala constituents: *Emblica officinalis*, *Terminalia belerica* and *Terminalia chebula*. J. Pharmacogn. Phytochem..

[36] Peterson C.T., Denniston K., Chopra D. (2017). Therapeutic uses of triphala in ayurvedic medicine. J. Altern. Complement. Med..

[37] López-García J., Lehocký M., Humpolíček P., Sáha P. (2014). HaCaT keratinocytes response on antimicrobial atelocollagen substrates: Extent of cytotoxicity, cell viability and proliferation. J. Funct. Biomater..

[38] Wu P.-C., Su C.-H., Cheng F.-Y., Weng J.-C., Chen J.-H., Tsai T.-L., Yeh C.-S., Su W.-C., Hwu J.R., Tzeng Y. (2008). Modularly assembled magnetite nanoparticles enhance in vivo targeting for magnetic resonance cancer imaging. Bioconjug. Chem..

[39] Aborehab N.M., Osama N. (2019). Effect of Gallic acid in potentiating chemotherapeutic effect of Paclitaxel in HeLa cervical cancer cells. Cancer Cell Int..

[40] Sharma J., Al-Omran A., Parvathy S. (2007). Role of nitric oxide in inflammatory diseases. Inflammopharmacology.

[41] Kim S.-H., Jun C.-D., Suk K., Choi B.-J., Lim H., Park S., Lee S.H., Shin H.-Y., Kim D.-K., Shin T.-Y. (2006). Gallic acid inhibits histamine release and pro-inflammatory cytokine production in mast cells. Toxicol. Sci..

[42] Oyawaluja B., Oyawaluja A., Babasanmi J., Soneye O. (2019). Phytochemistry and antioxidant assays of *Entandrophragma angolense* (Welw) C. DC.(meliaceae) using DPPH and nitric oxide free radical scavenging methods. Niger. J. Pharm. Res..

[43] Afsar T., Razak S., Shabbir M., Khan M.R. (2018). Antioxidant activity of polyphenolic compounds isolated from ethyl-acetate fraction of *Acacia hydaspica* R. Parker. Chem. Cent. J..

[44] Kalaiselvan S., Rasool M.K. (2015). The anti-inflammatory effect of triphala in arthritic-induced rats. Pharm. Biol..

[45] Parveen R., Shamsi T.N., Singh G., Athar T., Fatima S. (2018). Phytochemical analysis and in-vitro biochemical characterization of aqueous and methanolic extract of Triphala, a conventional herbal remedy. Biotechnol. Rep..

[46] Conte R., Marturano V., Peluso G., Calarco A., Cerruti P. (2017). Recent advances in nanoparticle-mediated delivery of anti-inflammatory phytocompounds. Int. J. Mol. Sci..

[47] Kapoor M., Cloyd J.C., Siegel R.A. (2016). A review of intranasal formulations for the treatment of seizure emergencies. J. Control. Release.

[48] Hinchcliffe M. (1996). The Novel Application of Chitosan for the Intranasal Delivery of Insulin. Doctoral Dissertation.

[49] Behl C., Pimplaskar H., Sileno A., Demeireles J., Romeo V. (1998). Effects of physicochemical properties and other factors on systemic nasal drug delivery. Adv. Drug Deliv. Rev..

[50] Salatin S., Alami-Milani M., Daneshgar R., Jelvehgari M. (2018). Box–Behnken experimental design for preparation and optimization of the intranasal gels of selegiline hydrochloride. Drug Dev. Ind. Pharm..

[51] Chen X., Zhi F., Jia X., Zhang X., Ambardekar R., Meng Z., Paradkar A.R., Hu Y., Yang Y. (2013). Enhanced brain targeting of curcumin by intranasal administration of a thermosensitive poloxamer hydrogel. J. Pharm. Pharmacol..

[52] Wang M., Ma X., Zong S., Su Y., Su R., Zhang H., Liu Y., Wang C., Li Y. (2024). The prescription design and key properties of nasal gel for CNS drug delivery: A review. Eur. J. Pharm. Sci..

[53] González N.N., Rassu G., Cossu M., Catenacci L., Sorrenti M.L., Cama E.S., Serri C., Giunchedi P., Gavini E. (2024). A thermosensitive chitosan hydrogel: An attempt for the nasal delivery of dimethyl fumarate. Int. J. Biol. Macromol..

[54] Ivanova N., Ermenlieva N., Simeonova L., Vilhelmova-Ilieva N., Bratoeva K., Stoyanov G., Andonova V. (2024). In Situ Gelling Behavior and Biopharmaceutical Characterization of Nano-Silver-Loaded Poloxamer Matrices Designed for Nasal Drug Delivery. Gels.

[55] Majithiya R.J., Ghosh P.K., Umrethia M.L., Murthy R.S. (2006). Thermoreversible-mucoadhesive gel for nasal delivery of sumatriptan. AAPS Pharmscitech.

[56] Hadidi M., Liñán-Atero R., Tarahi M., Christodoulou M.C., Aghababaei F. (2024). The potential health benefits of gallic acid: Therapeutic and food applications. Antioxidants.

[57] Marttin E., Schipper N.G., Verhoef J.C., Merkus F.W. (1998). Nasal mucociliary clearance as a factor in nasal drug delivery. Adv. Drug Deliv. Rev..

[58] Nair A.B., Chaudhary S., Shah H., Jacob S., Mewada V., Shinu P., Aldhubiab B., Sreeharsha N., Venugopala K.N., Attimarad M. (2022). Intranasal delivery of darunavir-loaded mucoadhesive in situ gel: Experimental design, in vitro evaluation, and pharmacokinetic studies. Gels.

[59] Lin H., Gebhardt M., Bian S., Kwon K.A., Shim C.-K., Chung S.-J., Kim D.D. (2007). Enhancing effect of surfactants on fexofenadineĚHCl transport across the human nasal epithelial cell monolayer. Int. J. Pharm..

[60] Phongpradist R., Thongchai W., Thongkorn K., Lekawanvijit S., Chittasupho C. (2022). Surface modification of curcumin microemulsions by coupling of KLVFF peptide: A prototype for targeted bifunctional microemulsions. Polymers.

[61] Phongpradist R., Jiaranaikulwanitch J., Thongkorn K., Lekawanvijit S., Sirilun S., Chittasupho C., Poomanee W. (2023). KLVFF Conjugated Curcumin Microemulsion-Based Hydrogel for Transnasal Route: Formulation Development, Optimization, Physicochemical Characterization, and Ex Vivo Evaluation. Gels.

[62] Cevher E., Taha M., Orlu M., Araman A. (2008). Evaluation of mechanical and mucoadhesive properties of clomiphene citrate gel formulations containing carbomers and their thiolated derivatives. Drug Deliv..

[63] Basu S., Maity S. (2012). Preparation and characterisation of mucoadhesive nasal gel of venlafaxine hydrochloride for treatment of anxiety disorders. Indian J. Pharm. Sci..

[64] Basu S., Bandyopadhyay A.K. (2011). Characterization of mucoadhesive nasal gels containing midazolam hydrochloride prepared from *Linum usitatissimum* L. mucilage. Braz. J. Pharm. Sci..

[65] Saudagar R.B., Deore S.B. (2016). In-Situ Nasal Gel Drug Delivery: An Overview. Res. J. Pharm. Dos. Forms Technol..

[66] Dawre S., Waghela S., Saraogi G. (2022). Statistically designed vitamin D3 Encapsulated PLGA microspheres dispersed in thermoresponsive in-situ gel for nasal delivery. J. Drug Deliv. Sci. Technol..

[67] Tichota D.M., Silva A.C., Sousa Lobo J.M., Amaral M.H. (2014). Design, characterization, and clinical evaluation of argan oil nanostructured lipid carriers to improve skin hydration. Int. J. Nanomed..

[68] Çelik Y.S., Örenli B., Al-Mohaya M., Mesut B., Özsoy Y. (2023). Nasal in situ gels as a drug delivery system: An overview of literature and clinical studies. J. Res. Pharm..

[69] Hanafy N.A., Leporatti S., El-Kemary M.A. (2019). Mucoadhesive hydrogel nanoparticles as smart biomedical drug delivery system. Appl. Sci..

[70] Makade C.S., Shenoi P.R., Bhongade B.A., Shingane S.A., Ambulkar P.C., Shewale A.M. (2024). Estimation of MBC: MIC Ratio of Herbal Extracts against Common Endodontic Pathogens. J. Pharm. Bioallied Sci..

[71] Ahluwalia V., Elumalai S., Kumar V., Kumar S., Sangwan R.S. (2018). Nano silver particle synthesis using *Swertia paniculata* herbal extract and its antimicrobial activity. Microb. Pathog..

[72] Biradar Y.S., Jagatap S., Khandelwal K., Singhania S.S. (2008). Exploring of antimicrobial activity of Triphala Mashi—An ayurvedic formulation. Evid.-Based Complement. Altern. Med..

[73] Osman N., Devnarain N., Omolo C.A., Fasiku V., Jaglal Y., Govender T. (2022). Surface modification of nano-drug delivery systems for enhancing antibiotic delivery and activity. Wiley Interdiscip. Rev. Nanomed. Nanobiotechnol..

[74] Raza A., Sime F.B., Cabot P.J., Maqbool F., Roberts J.A., Falconer J.R. (2019). Solid nanoparticles for oral antimicrobial drug delivery: A review. Drug Discov. Today.

[75] Gontijo V.S., Espuri P.F., Alves R.B., de Camargos L.F., dos Santos F.V., de Souza Judice W.A., Marques M.J., Freitas R.P. (2015). Leishmanicidal, antiproteolytic, and mutagenic evaluation of alkyltriazoles and alkylphosphocholines. Eur. J. Med. Chem..

[76] Silva V.A., Gonçalves G.F., Pereira M.S., Gomes I.F., Freitas A.F., Diniz M.F., Pessôa H.L. (2013). Assessment of mutagenic, antimutagenic and genotoxicity effects of Mimosa tenuiflora. Rev. Bras. Farmacogn..

[77] Borges F.F.V., Silva C.R., Véras J.H., Cardoso C.G., da Cruz A.D., Chen L.C. (2016). Antimutagenic, antigenotoxic, and anticytotoxic activities of *Silybum marianum* [L.] Gaertn assessed by the *Salmonella* mutagenicity assay (Ames test) and the micronucleus test in mice bone marrow. Nutr. Cancer.

[78] Kaur S., Arora S., Kaur K., Kumar S. (2002). The in vitro antimutagenic activity of Triphala—An Indian herbal drug. Food Chem. Toxicol..

[79] Buschini A., Ferrarini L., Franzoni S., Galati S., Lazzaretti M., Mussi F., Northfleet de Albuquerque C., Maria Araújo Domingues Zucchi T., Poli P. (2009). Genotoxicity revaluation of three commercial nitroheterocyclic drugs: Nifurtimox, benznidazole, and metronidazole. J. Parasitol. Res..

[80] Makhafola T.J., Elgorashi E.E., McGaw L.J., Verschaeve L., Eloff J.N. (2016). The correlation between antimutagenic activity and total phenolic content of extracts of 31 plant species with high antioxidant activity. BMC Complement. Altern. Med..

[81] Hour T.-C., Liang Y.-C., Chu I.-S., Lin J.-K. (1999). Inhibition of eleven mutagens by various tea extracts, (−) epigallocatechin-3-gallate, gallic acid and caffeine. Food Chem. Toxicol..

[82] Chittasupho C., Athikomkulchai S., Samee W., Na Takuathung M., Yooin W., Sawangrat K., Saenjum C. (2023). Phenylethanoid Glycoside-Enriched Extract Prepared from *Clerodendrum chinense* Leaf Inhibits A549 Lung Cancer Cell Migration and Apoptosis Induction through Enhancing ROS Production. Antioxidants.

[83] Singh S., Chidrawar V.R., Hermawan D., Nwabor O.F., Olatunde O.O., Jayeoye T.J., Samee W., Ontong J.C., Chittasupho C. (2023). Solvent-assisted dechlorophyllization of *Psidium guajava* leaf extract: Effects on the polyphenol content, cytocompatibility, antibacterial, anti-inflammatory, and anticancer activities. S. Afr. J. Bot..

[84] Chittasupho C., Kengtrong K., Chalermnithiwong S., Sarisuta N. (2020). Anti-angiogenesis by dual action of R5K peptide conjugated itraconazole nanoparticles. AAPS PharmSciTech.

[85] Angsusing J., Singh S., Samee W., Tadtong S., Stokes L., O’connell M., Bielecka H., Toolmal N., Mangmool S., Chittasupho C. (2024). Anti-inflammatory activities of Yataprasen Thai traditional formulary and its active compounds, beta-amyrin and stigmasterol, in RAW264. 7 and THP-1 cells. Pharmaceuticals.

[86] Sherafudeen S.P., Vasantha P.V. (2015). Development and evaluation of in situ nasal gel formulations of loratadine. Res. Pharm. Sci..

[87] Xu X., Shen Y., Wang W., Sun C., Li C., Xiong Y., Tu J. (2014). Preparation and in vitro characterization of thermosensitive and mucoadhesive hydrogels for nasal delivery of phenylephrine hydrochloride. Eur. J. Pharm. Biopharm..

[88] Punvittayagul C., Sringarm K., Chaiyasut C., Wongpoomchai R. (2014). Mutagenicity and antimutagenicity of hydrophilic and lipophilic extracts of Thai northern purple rice. Asian Pac. J. Cancer Prev..

